# Association between gestational and childhood exposures to organophosphate esters and social skill, problem behavior among adolescents

**DOI:** 10.1016/j.envres.2026.124553

**Published:** 2026-04-20

**Authors:** Jagadeesh Puvvula, Joseph M. Braun, Kimberly Yolton, Kim M. Cecil, Antonia M. Calafat, Maria Ospina, Whitney Fitts, Kelli M. Williams, Weili Yang, Ann M. Vuong, Zana Percy, Bruce Lanphear, Aimin Chen

**Affiliations:** a Department of Biostatistics, Epidemiology and Informatics, Perelman School of Medicine, University of Pennsylvania, Philadelphia, PA, USA; b Department of Epidemiology, Brown University, Providence, RI, USA; c Department of Environmental and Public Health Sciences, University of Cincinnati College of Medicine, Cincinnati, OH, USA; d Department of Pediatrics, Cincinnati Children’s Hospital Medical Center, University of Cincinnati College of Medicine, Cincinnati, OH, USA; e Department of Radiology, Cincinnati Children’s Hospital Medical Center, University of Cincinnati College of Medicine, Cincinnati, OH, USA; f National Center for Environmental Health, U.S. Centers for Disease Control and Prevention, Atlanta, GA, USA; g Division of Neurology, Children’s Hospital of Philadelphia, Philadelphia, PA, USA; h Department of Epidemiology and Biostatistics, School of Public Health, University of Nevada Las Vegas, Las Vegas, NV, USA; i Faculty of Health Sciences, Simon Fraser University, Burnaby, BC, Canada; j Child and Family Research Institute, BC Children’s Hospital, Vancouver, BC, Canada

**Keywords:** Organophosphate esters, Behavior, Social skills, And repeated exposures

## Abstract

Early life exposure to organophosphate esters (OPEs), a class of flame retardants and plasticizers, may be associated with adverse neurobehavioral outcomes. Due to OPEs’ relatively short biological half-life in humans, it is important to evaluate exposure-response associations using repeated measures during gestation and childhood. We examined the associations between OPE biomarkers and social skill/problem behavior using data from 236 caregiver-adolescent dyads in the Health Outcomes and Measures of the Environment (HOME) Study. We included urinary concentrations of four OPE biomarkers [bis-2-chloroethyl-phosphate (BCEP), bis(1,3-dichloro-2-propyl)-phosphate (BDCIPP), di-n-butyl-phosphate (DNBP), diphenyl-phosphate (DPHP)] measured up to 9 times (3 in gestation and 6 in childhood) and calculated cumulative exposure measures during gestation, childhood, and the lifetime. Social and behavioral outcomes at age 12 years were assessed using the Social Skills Improvement System (SSiS) completed by adolescents and caregivers. We used quantile g-computation regression to evaluate joint associations between the OPE biomarker mixture and SSiS scores, while adjusting for covariates. Overall, we observed null associations between OPE biomarkers and adolescent social skill/problem behavior scores, but the effect measure modification by adolescent sex was statistically significant. Every quartile increase in childhood/lifetime OPE biomarker mixture was associated with improved caregiver-reported problem behavior among females (*Ψ*_*childhood*_ −3.44 (95%CI: −6.05, −0.84); *Ψ*_*lifetime*_ −3.82 (95%CI: −6.43, −1.22)). Every quartile increase in lifetime OPE biomarker mixture was associated with impaired social skill scores in males (*Ψ*_*lifetime*_ −3.59 (95%CI: −6.79, −0.40). Further studies with a larger sample size will help provide additional insights into OPE exposures early in life and subsequent sex-specific associations with adolescent social skill/problem behavior scores.

## 1. Introduction

Organophosphate esters (OPEs) are phosphoric acid esters containing alkyl or aryl chains, some of which are halogenated (Blum et al., 2019). OPEs were introduced as substitutes for brominated flame retardants, which were phased out due to environmental persistence and potential adverse effects on human health (Sharkey et al., 2020). Among the OPEs, those with chlorinated alkyl chains, such as tris(2-chloroethyl) phosphate (TCEP) and tris(1,3-dichloro-2-propyl) phosphate (TDCIPP), are commonly used as flame retardants in furniture, electronic devices, and construction materials. Tri-n-butyl phosphate (TnBP) includes an n-butyl alkyl chain but without chlorine used as a flame retardant and plasticizer, while OPEs with aryl chains, such as triphenyl phosphate (TPHP), are used as plasticizers (van der Veen and de Boer, 2012; Cequier et al., 2014). OPEs are not chemically bound to products, which allows them to leach into the environment (Marklund et al., 2003; Reemtsma et al., 2008).

Due to the widespread use of OPEs in consumer products and building materials, human exposure may occur through inhalation, ingestion, or dermal absorption of indoor dust (Peng et al., 2023), as well as through diet (Marklund et al., 2003; He et al., 2015; Wu et al., 2016; Nguyen et al., 2024). OPE metabolites were detected in over 60% of participants (age ≥6 years) in the U.S. National Health and Nutrition Examination Survey, indicating chronic exposures (Ospina et al., 2018). For children, exposure to OPEs is primarily from indoor dust (Wei et al., 2015). Phillips et al. identified OPEs in over 80% of house dust and child hand wipe samples (central North Carolina), with OPE metabolites detected in more than 94% of child urine samples, and reported associations between hand wipe OPE concentrations and urinary OPE metabolite concentrations, implying potential exposure through hand-to-mouth activity (Phillips et al., 2018). Because of OPEs’ relatively short biological half-lives, previous studies reported low to moderate within-person correlations between urinary OPE metabolite concentrations among children who provided multiple spot urine samples over time (Louis et al., 2022; Yang et al., 2024; Wang et al., 2019). Thus, there is a need to include repeated exposure biomarker measurements to identify windows of susceptibility and potentially minimize exposure misclassification bias.

Exposure to OPEs has been linked to adverse neurobehavioral outcomes, potentially stemming from their interference with neurotransmission and the endocrine system (Patisaul et al., 2021a). OPE metabolites potentially interact with neurotransmitters [N-methyl-D-aspartate (NMDA), gamma-aminobutyric acid (GABA), acetylcholine], nuclear receptors (estrogen, androgen, peroxisome proliferator-activated receptor γ), and voltage-gated chloride channels (Patisaul et al., 2021a). Several epidemiologic studies (Hall et al., 2023; Percy et al., 2021, 2023; Doherty et al., 2019; Vuong et al., 2024; Hernandez-Castro et al., 2023; Castorina et al., 2017) have examined the associations between gestational OPEs and behavioral outcomes, reporting inconsistent findings. Only Lipscomb et al. explored cross-sectional associations between childhood exposure to OPEs and social behavior among preschoolers and reported OPEs are associated with less responsibility and more externalizing behavioral problems (Lipscomb et al., 2017). Prior studies have assessed OPE biomarkers at relatively few time points, focusing on exposure during gestation or childhood. Additionally, adolescent behavior and social skills are key to socio-emotional development and may play a critical role in future job function and personal life-related outcomes (Ferris et al., 2001; Witt and Ferris, 2003). We aimed to assess associations between gestational and childhood urinary OPE biomarkers and problem behaviors and social skills among adolescents.

## 2. Methods

### 2.1. Study participants

We utilized data from the Health Outcomes and Measures of the Environment (HOME) Study, a longitudinal pregnancy and birth cohort. The study enrolled pregnant women between March 2003 and January 2006. Eligible participants were residing in the Cincinnati, Ohio metropolitan area, fluent in English, aged ≥18 years, at 13–19 weeks of gestation, not living in a mobile or trailer home, residing in a home built before 1978, not planning to relocate from the Cincinnati area within the next year, HIV-negative, not taking thyroid or seizure medications, and not diagnosed with bipolar disorder, schizophrenia, diabetes, or cancer requiring radiation or chemotherapy (Braun et al., 2017). Among the 468 enrolled participants, 389 delivered singletons, 9 delivered sets of twins, and 3 experienced stillbirths. Study visits were conducted at the 16th and 26th weeks of gestation, within 48 h of delivery, and around the child’s 1st, 2nd, 3rd, 4th, 5th, 8th, and 12th birthdays. The pregnant women provided consent for their own participation and for their children’s participation. The participating children provided assent at the 12-year follow-up visit. The study protocol was approved by the institutional review board (IRB) at the Cincinnati Children’s Hospital Medical Center (CCHMC). The Centers for Disease Control and Prevention (CDC) deferred to CCHMC as the IRB of record. The analysis of the age 12-year visit samples at the CDC was determined not to constitute engagement in human subjects’ research.

### 2.2. Urinary OPE biomarkers

We collected maternal urine samples in polypropylene specimen cups at approximately the 16th and 26th week of gestation and within 48 h of delivery. The child’s spot urine samples were collected using Kendall abdominal pads placed in a diaper for non-toilet-trained children, using a pad-lined training toilet for children in toilet training, and in propylene specimen cups for toilet-trained children in the 1st, 2nd, 3rd, 5th, 8th, and 12th years of the child’s life. All urine samples were stored at −20 °C and subsequently shipped frozen overnight on dry ice to the CDC’s laboratory. The urinary concentrations of four OPE metabolites, bis-2-chloroethyl phosphate (BCEP; parent TCEP), bis(1,3-dichloro-2-propyl) phosphate (BDCIPP; parent TDCIPP), di-n-butyl phosphate (DNBP; parent TnBP), and diphenyl phosphate (DPHP; parent TPHP), were measured using solid phase extraction and high-performance liquid chromatography isotope dilution tandem mass spectrometry (Jayatilaka et al., 2017, 2019). The detection limit (LOD) of all OPE biomarkers was 0.1 μg/L. The method precision was calculated as the coefficient of variation (%CV) obtained from the analysis of 2 distinct quality control materials prepared in duplicates during 20 consecutive days, the inter-day CV ranged between 2.1 and 9.8%, depending on the OPE metabolite, which is within the U.S.-FDA recommended limits (Jayatilaka et al., 2019). Method accuracy, calculated by the spike recovery at three distinct (0.5, 5, 20 μg/L) concentrations, was 90–118%, depending on the analyte (Jayatilaka et al., 2019).

### 2.3. Social Skills Improvement System (SSiS) assessment

The Social Skills Improvement System (SSiS) was employed to assess social skills and problematic behaviors among adolescents at 12 years (Table S1). (Gresham and Elliott, 2008) The SSiS scale is designed to quantify the strengths and difficulties in the acquisition, performance, and challenging behaviors that impact social interactions among adolescents. The social skills sub-scale covers elements such as communication (pragmatic skill), cooperation (helping others, sharing, and compliance), assertion (requesting, initiating, and responding appropriately), responsibility (showing respect for property and communicating with adults), empathy (showing concern for others’ feelings and perspectives), engagement (joining and inviting others to join activities, making friends), and self-control (responding appropriately to conflict and compromising) (Channell et al., 2023). The problem behavior segment covers: externalizing (verbal or physical aggression), bullying (hurting others physically or emotionally, excluding others), hyperactivity/inattention (fidgety, impulsive, and easily distracted), and internalizing (anxious, sad, or lonely) (Channell et al., 2023). Reynolds et al. reported a higher internal consistency for the caregiver-reported composite (α 0.94–0.96) and subscale scores (α 0.83–0.87) and a high correlation with the Behavior Assessment System for Children (BASC-2) scores (SSiS-social skills and BASC-adaptive skills r = 0.62–0.66; SSiS-problem behaviors and BASC-2-behavioral symptom index r = 0.80–0.82) (Channell et al., 2023; Reynolds, 2010).

In this study, adolescent participants completed the SSiS instrument through self-report using the student form, while caregivers completed the parent form. We calculated age-standardized composite scores (mean of 100, SD of 15) for the adolescent’s social skills and problem behavior, as evaluated by both the adolescent and their caregiver, using publisher-supplied U.S. normative data. A lower social skills score indicated a deficit in the adolescent’s social skills, whereas a higher problematic behavior score indicated more problematic behavior. Among the 389 study participants who delivered singletons, 256 participated in the 12-year follow-up visit.

### 2.4. Covariates

Based on prior literature, we selected covariates for minimal adjustment (maternal education, race, and household income) and additional precision variables and co-exposures [maternal factors (age, depression, marital status, relationship frustration score, cotinine, lead), and child sex], while exploring the association between urinary OPE biomarker concentrations and SSiS scores (Fig. S1). (Hartley et al., 2022) All the covariates, except for maternal age at delivery, were measured at the child’s 12th-year visit. Among the included covariates, adolescent sex, race (White persons and Other), caregiver marital status (married and unmarried), maternal education (less than bachelor’s degree and bachelor’s degree or more), maternal age at delivery, and maternal depression (Beck Depression Inventory-II score: minimal depression <13 and greater than minimal depression ≥13) were included as categorical variables (Beck et al., 1996).

Furthermore, household income, blood lead levels (μg/dL), serum cotinine concentrations (μg/L), and Relational Frustration scores reported by the caregiver were included as continuous covariates (Kamphaus and Reynolds, 2006). Blood lead levels were quantified using inductively coupled plasma mass spectrometry with an LOD of 0.18 μg/dL, and serum cotinine concentrations were measured using high-performance liquid chromatography isotope dilution tandem mass spectrometry with an LOD of 0.015 μg/L (Nixon et al., 1999; DeLorenze et al., 2002) Blood lead and serum cotinine concentrations measured at 16th and 26th weeks of gestation were averaged to reflect the gestational period and were used in this analysis.

### 2.5. Statistical analysis

We included 236 study participants with SSiS scores and OPE biomarker measurements available from at least one of the 9 time points. Since a relatively high proportion of DNBP biomarker concentrations during the child visit at years 1 (79%) and 2 (98%) could not be reported due to the presence of interfering substances (Table S3), we excluded DNBP concentrations collected at the child’s year 1&2 visits from the analysis as illustrated in Fig. S2.

OPE biomarker concentrations below the LOD were imputed (processed by biomarker and visit) using the left-truncated log-normal distribution (Lubin et al., 2004; Mersmann et al., 2023). Furthermore, we measured urine-specific gravity with the Atago (PAL-10S) handheld refractometer (ATAGO Co., Tokyo, Japan). Then, we standardized the potential inter-individual variance in OPE biomarker concentrations per visit by applying the formula below (Duty et al., 2005).


$$STD-{\text{OPE concentration}}_{\text{urine}}=OPE_{i}\frac{\left(SG_{m}-1\right)}{\left(SG_{i}-1\right)}$$

STD-OPE concentration represents the urinary OPE biomarker concentrations standardized using urine specific gravity, OPE_i_ is the urinary OPE biomarker concentration measured for ith observation, SG_m_ represents the median urinary specific gravity of the participant pool, and SG_i_ is the urinary specific gravity of the ith participant.

We estimated cumulative measures of OPE biomarkers by calculating the area under the time-concentration curve (AUC) using the trapezoid method. The AUC values were calculated using specific gravity standardized OPE biomarker concentrations during gestation (using measurements from the 16, 26 weeks of gestation, and delivery), childhood (measurements from 1, 2, 3, 5, 8, and 12 years visit of the child), and lifetime (measurements from gestation and childhood combined) (Table S2 and S3). We calculated intraclass correlation coefficients (ICCs) from OPE metabolite concentrations (log_2_-transformed and specific gravity-standardized) using linear mixed-effect models. The ICCs are interpreted as ≤0.4 indicates poor, 0.4–0.75 indicates fair to good, and ≥0.75 indicates excellent (Rosner, 2006). Estimated AUC values were then divided by the length of the measurements and included for further analysis.

#### 2.5.1. Joint association between OPE biomarkers and adolescent SSiS scores

To identify susceptible periods of vulnerability to the OPE biomarker mixture, we applied quantile g-computation regression to estimate the association between urinary OPE biomarker concentrations and SSiS scores stratified by three time points (gestation, childhood, and lifetime) (Keil et al., 2020; Keil, 2025a). We performed a bootstrap version of the quantile g-computation regression using 400 bootstrap samples to assess potential uncertainty. Furthermore, we assessed effect measure modification by adolescent sex on the association between OPE biomarker mixtures and SSiS scores using a quantile g-computation framework, considering interaction terms with p-values <0.1 as statistically significant (Keil, 2025b). Additionally, we assessed the joint associations between the OPE biomarker mixture and SSiS scores stratified by adolescent sex. Quantile g-computation regression analyses were performed by transforming the OPE biomarker mixture to a quartile scale. Effect estimates from quantile g-computation are interpreted as associations per quartile increase in the OPE biomarker mixture.

#### 2.5.2. Sensitivity analysis: association between single OPE biomarker and adolescent SSiS scores

We used multiple linear regression to assess the associations between individual log_2_-transformed OPE biomarker AUC values and adolescent SSiS scores, adjusting for the same covariates as in the joint association model. The effect estimates correspond to every two-fold increase in OPE biomarker concentrations.

## 3. Results

The median age of pregnant women was 29 years [interquartile range (IQR): 24.75, 33.00] at delivery (Table 1). At the 12-year visit, the majority (65.68%) of the caregivers were married or living with a partner; 52.12% completed their bachelor’s education or above, and lived in a household with a median household income of $75,000 (IQR: 35000, 145,000), and most (85.59%) reported minimal depression. The majority of children born into the study (55.93%) were female, and 56.36% identified as White.

Among the OPE biomarkers assessed in this study, DPHP was detected at a higher frequency, with a range between 97 and 100% across the follow-ups, and DNBP had a relatively lower detection frequency (45–83% excluding child visit-1,2) (Table S4). Across the follow-up visits, the OPE biomarkers were detected at a relatively higher frequency during the child follow-up visits at 3 and 8 years. Across the OPE biomarker measurement periods, we observed ICCs of greater magnitude (range: 0.07–0.35) during gestation than during childhood or the lifetime (Table S5). We observed relatively stronger ICCs for BDCIPP compared with other OPE biomarkers. These OPE biomarker ICCs showed poor temporal consistency in OPE measures (Fig. S3). The area under the curve measures of OPE biomarkers (BCEP, BDCIPP, and DPHP) was higher during childhood than during the gestational period (Table 2). Overall, we observed positive correlations between the self-reported (adolescent) and caregiver-reported SSiS scores (r_social_-_skill_:0.26; r_problem behavior_:0.43) (Fig. S4). We observed a relatively weak correlation between self and caregiver reported SSiS scores among males (r_social-skill_:0.13; r_problem behavior_:0.34), compared to females (r_social-skill_:0.31; r_problem behavior_:0.50).

### 3.1. Associations between OPE biomarkers and problem behavior scores

#### 3.1.a. Adolescent-reported problem behavior:

**Fig. 1.**
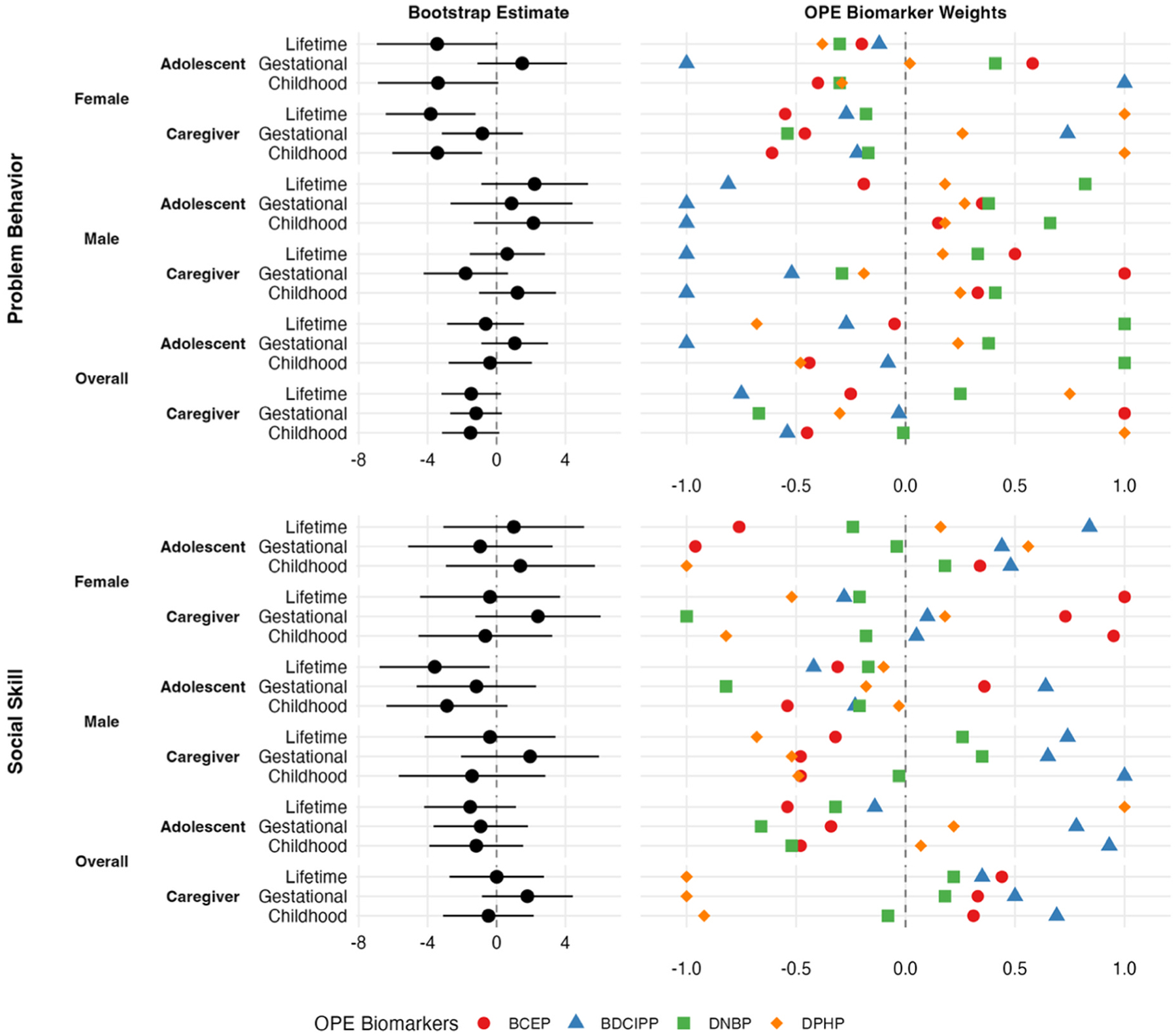
Joint association between gestational, childhood, and lifetime measures of urinary OPE metabolites with social skill and problem behaviors among adolescents. Estimates presented here are generated using boot strap version (400 bootstrap samples) and biomarker weights from the non-bootstrap version of the quantile g-computation regression. Associations adjusted for adolescent sex (for overall analysis), race, caregiver marital status, maternal education, maternal age at delivery, maternal depression, household income, blood lead, serum cotinine, and Relational Frustration scores.

Overall, we observed null associations between the OPE biomarker mixture and self-reported problem behavior among adolescents (Fig. 1; Table S6). A quartile increase in gestational OPE biomarker mixture was associated with a 1.06-point increase in problem behavior (95% CI: −0.88, 2.99; EMM_sex_:0.55) (Table S7). However, childhood/lifetime AUC of OPE biomarker mixtures showed null associations with self-reported problem behavior [*Ψ*_*childhood*_: −0.37 (95%CI: −2.78, 2.05) & *Ψ*_*lifetime*_: − 0.63 (95%CI: −2.86, 1.60)].

Despite the overall null associations, effect measure modification by adolescent sex (EMMchildhood:0.02 & EMMlifetime:0.03) was statistically significant. Associations by adolescent sex demonstrated diverging patterns, where the association was protective among females [*Ψ*_*childhood*_: −3.40 (95%CI: −6.89, 0.10) & *Ψ*_*lifetime*_: −3.45 (95%CI: −6.95, 0.05], but adverse among males [*Ψ*_*childhood*_: 2.14 (95%CI: −1.32, 5.60) & *Ψ*_*lifetime*_: 2.21 (95%CI: 0.88, 5.31]. Among females, BCEP, DNBP, and DPHP consistently contributed to the protective trend, consistent with our single-chemical analysis, in which all OPE biomarkers showed protective trends during childhood/lifetime (Table S8). Among males, DNBP contributed most to the increased problem behavior trends, which aligned with our single chemical analysis.

#### 3.1.b. Caregiver-reported problem behavior:

We observed protective trends between the OPE biomarker mixture and overall caregiver-reported problem behavior scores. Effect modification by adolescent sex was statistically significant between childhood and lifetime (EMM-_childhood_:0.01 & EMM_lifetime_:0.01) OPE biomarkers and caregiver-reported problem behavior. As with self-reported problem behavior, we observed divergent trends between males and females. Among females, every quartile increase in childhood OPE biomarker mixture was associated with a 3.44-point decrease (95%CI: −6.05, −0.84) in caregiver-reported problem behavior scores, while lifetime AUC of OPEs was associated with a 3.82-point decrease (95% CI: −6.43, −1.22). BCEP and BDCIPP contributed most to these protective associations, and these trends were supported by our findings from the single-chemical biomarker analysis. In contrast, childhood/lifetime OPE biomarker mixtures showed a weak positive trend with caregiver-reported problem behavior among males [*Ψ*_*childhood*_: 1.22 (95%CI: −1.01, 3.45) & *Ψ*_*lifetime*_: 0.62 (95%CI: −1.56, 2.81)].

### 3.2. Associations between OPE biomarkers and social skill scores

#### 3.2.a. Adolescent-reported social skill:

Overall, the OPE biomarker mixture showed a decreasing trend with self-reported social skill scores. Effect modification by adolescent sex was statistically significant for associations between childhood (EMM_sex_:0.06) OPE mixtures and self-reported social skills. Similar to the problem behavior outcomes, we observed diverging trends between males and females, where females showed protective trends (*Ψ*_*childhood*_: 1.38 (95%CI: −2.94, 5.71) & *Ψ*_*lifetime*_: 1.00 (95%CI: −3.09, 5.09)). Among males, a quartile increase in lifetime OPE biomarker mixture was associated with a 3.59-point decrease (95%CI: −6.79, −0.40) in self-reported social skill score. All four OPE biomarkers synergistically contributed to this association; these associations remained non-significant while using the single biomarker approach.

#### 3.2.b. Caregiver-reported social skill:

We observed inconsistent trends between the OPE biomarker mixture and caregiver-reported social skills across the biomarker assessment windows. A quartile increase in gestational OPE mixtures was associated with a 1.79-point increase (95%CI: −0.85, 4.43) in caregiver-reported social skills. However, childhood/lifetime OPE biomarker associations with caregiver-reported social skill scores remained null [*Ψ*_*childhood*_: −0.47 (95%CI: −3.11, 2.16) & *Ψ*_*lifetime*_: 0.01 (95%CI: − 2.73, 2.75)]. Additionally, we did not observe effect modification by adolescent sex.

## 4. Discussion

In this study, we evaluated associations between gestational and childhood OPE biomarker concentrations in urine and adolescent/caregiver-reported SSiS scores. We observed an overall null association between OPE biomarker mixtures (at gestation, childhood, and lifetime) and SSiS scores among adolescents. However, adolescent sex modified the association between childhood/lifetime OPE biomarker mixtures and adolescent-reported SSiS scores. Although individual sex-specific associations were modest in effect size, there were divergent patterns in associations between OPEs and problem behavior by adolescent sex, where females showed protective trends, and males showed an adverse trend. Among females, childhood/lifetime OPE biomarker mixture was associated with a decrease in caregiver-reported problem behavior scores. Additionally, the lifetime OPE biomarker mixture was associated with a decrease in self-reported social skills among males.

Limited studies (Hall et al., 2023; Percy et al., 2021, 2023; Doherty et al., 2019; Vuong et al., 2024; Hernandez-Castro et al., 2023; Castorina et al., 2017; Lipscomb et al., 2017) have explored the associations between OPE biomarkers and behavioral outcomes among children, and these studies (3 of 8 were from the HOME Study (Percy et al., 2023; Percy et al., 2021; Vuong et al., 2024)) primarily focused on gestational exposures. Findings from the Pregnancy, Infection, and Nutrition (PIN) Study suggested that maternal OPE biomarkers (BDCIPP and DPHP) were associated with increased maternal-reported Behavioral Symptom Index scores and showed a protective trend for DPHP and internalizing behaviors among children aged 3 years (Doherty et al., 2019). In the Center for the Health Assessment of Mothers and Children of Salinas (CHAMACOS) Study, gestational BDCIPP and DPHP showed null associations with self-/teacher-reported BASC-2 scores, while iso-propylphenyl phenyl phosphate (ip-PPP) with a 2.4 points higher (95% CI: 0.1, 4.7) maternal-reported BASC-2 hyperactivity scores among children of 7 years; whereas DPHP showed marginal protective trend with hyperactivity scores (Castorina et al., 2017). A cross-sectional study using passive samplers identified that OPEs were associated with less responsible behavior and higher teacher-rated externalizing behavioral problems among preschool-aged children (Lipscomb et al., 2017). Hernandez et al. found that BCIPP measured in the third trimester of pregnancy was associated with higher internalizing scores in children at 3 years using the Child Behavior Checklist (CBCL), and DBCIPP showed a protective trend (Hernandez-Castro et al., 2023). Furthermore, a study assessed OPE metabolite concentrations during the second trimester (17 weeks’ gestation) and executive function, suggesting inconsistent trends between DPHP, DNBP, and inhibition, working memory, and emotional control among children aged 2–5 years (Hall et al., 2023). Prior studies based on the HOME Study participants reported associations between OPE biomarkers at delivery and higher behavioral problems among children (ages 3 and 8 years) using the BASC-2 (Percy et al., 2023) and worsened executive function among adolescents using the BRIEF-2 (Vuong et al., 2024). We observed protective trends between OPEs and the SSiS score among females, consistent with prior literature showing protective associations between gestational OPEs and child behavioral assessments (Hall et al., 2023; Doherty et al., 2019). OPEs are recognized as endocrine-disrupting and potentially neurotoxic, so a biological protective effect is unlikely; instead, protective estimates may reflect residual confounding (protective health behaviors or socioeconomic factors correlated with OPE exposure).

Although population-based studies yield inconsistent findings, mammalian studies indicate that OPEs may inhibit acetylcholine, GABA, neuropathy target esterase (NTE), and volatile-dependent chloride channels (Patisaul et al., 2021a). Potential pathways include OPE-mediated inhibition of acetylcholinesterase, leading to acetylcholine accumulation; and OPE-mediated dopamine antagonism, potentially altering neurotransmission and resulting in neurodevelopmental toxicity (Guignet et al., 2019; Oliveri et al., 2018). Additionally, OPE-mediated GABA antagonism could lead to the blockage of GABA-regulated chloride channels, affecting GABAergic neurotransmission (Gant et al., 1987). Furthermore, inhibition of the NTE enzyme could result in organophosphate-induced delayed neuropathy (Kobayashi et al., 2017; Richardson et al., 2020; Faria et al., 2018). OPEs may interfere with the hypothalamic–pituitary–gonadal (HPG) and hypothalamic–pituitary–adrenal (HPA) axes, altering the synthesis and regulation of gonadotropins and steroid hormones (estradiol, progesterone, testosterone) in a sex-dependent manner (Patisaul et al., 2021b). Preclinical studies demonstrated that OPE exposures alter hypothalamic gene expression, disrupt steroidogenesis in gonadal cells, leading to sex-specific changes in anxiety-related and socioemotional behaviors, supporting a hormonally mediated mechanism (Patisaul et al., 2021a; Baldwin et al., 2017; Li et al., 2025).

This study assessed associations between repeated OPE biomarkers and adolescent SSiS scores to identify potential patterns. Because OPE biomarkers have a relatively short half-life, repeated exposure assessments during gestation and childhood strengthen our findings by potentially minimizing exposure misclassification bias. Additionally, the data-rich environment from the HOME Study allowed us to adjust for several potential confounders.

Our study has certain limitations, including a limited sample size to consider analysis stratified by adolescent sex. The spot urine collection protocol for OPE biomarker quantification did not account for the time of sample collection (morning vs. evening), which may reflect in potential misclassification. Limiting the OPE exposure assessments to four metabolites may introduce residual bias due to the unmeasured OPE biomarkers, such as bis(2-butoxyethyl) phosphate.

## 5. Conclusion

We observed overall null associations between urinary OPE biomarkers and problem behavior/social skill scores among adolescents. However, we observed some evidence that sex modified these associations. Among females, we observed that the OPE biomarker mixture was associated with less caregiver-reported problem behavior scores. This finding was consistent with those from the single-chemical approach. Additionally, the lifetime measure of OPE biomarkers was associated with a decrease in self-reported social skills among males.

## Supplementary Material

1

## Figures and Tables

**Table 1:** Participant characteristics included in the analysis: HOME study (n = 236).

Variable	Value	Caregiver reported scores mean (SD)	
		Problem behavior^a^	Social skill^a^
Adolescent sex			
Male	104 (44.07)	96 (12)	97 (14)
Female	132 (55.93)	97 (13)	101 (16)
Adolescent race			
White	133 (56.36)	95 (11)	102 (13)
Other	103 (43.64)	99 (13)	96 (17)
Maternal marital status			
Married or living with a partner	155 (65.68)	96 (12)	101 (14)
Not-married and living alone	81 (34.32)	99 (12)	96 (17)
Maternal education			
Less than bachelor's degree	113 (47.88)	99 (13)	95 (16)
Bachelor's education or above	123 (52.12)	95 (11)	103 (13)
Maternal depression (BDI-II)			
Minimal (0–13)	202 (85.59)	95 (12)	100 (15)
Mild, moderate, or severe (>13)	34 (14.41)	104 (11)	94 (14)
Maternal age at delivery (years)*	29.00 (24.75, 33.00)	≤29: 98 (13) >29: 95 (11)	≤29: 97 (16) >29: 102 (13)
Relational frustration*	48.00 (41.00, 53.00)	≤48: 95 (12) >48: 99 (13)	≤48: 100 (15) >48: 98 (15)
Median household income*	$75,000 (35000, 145,000)	≤$75,000: 98 (13) >$75,000: 95 (11)	≤$75,000: 96 (17) >$75,000: 102 (12)
Maternal gestational serum cotinine (μg/L)*	0.04 (0.01, 0.25)	≤0.04: 94 (11) >0.04: 99 (13)	≤0.04: 102 (12) >0.04: 96 (17)
Maternal gestational blood lead level (μg/dL)*	0.66 (0.52, 0.81)	≤0.66: 95 (12) >0.66: 98 (12)	≤0.66: 101 (14) >0.66: 97 (16)
SSiS scores			
Problem behavior – Adolescent*	95 (86, 105)		
Problem behavior – Caregiver*	92 (86, 102)		
Social skills – Adolescent*	101 (88, 111)		
Social skills – Caregiver*	100 (89, 112)		

All the participant characteristics listed in this table were measured at the adolescent’s 12th year follow up visit. The table contains counts and percentages for categorical variables and median and interquartile ranges for continuous variables.

*- Continuous variables included in this table.

a Participant characteristics measured on a continuous scale were stratified using the median value to highlight potential differences in caregiver reported SSiS scores.

**Table 2:** Median (25th, 75th percentile) cumulative OPE biomarker measures (μg/L).

OPE biomarker	Visit		
	Gestational	Childhood	Lifetime
BCEP	0.72 (0.42, 1.41)	1.09 (0.57, 2.09)	1.23 (0.62, 2.33)
BDCIPP	0.84 (0.51, 1.51)	3.83 (1.92, 6.19)	3.59 (1.88, 5.87)
DNBP	0.22 (0.15, 0.37)	0.17 (0.11, 0.28)	0.21 (0.13, 0.34)
DPHP	2.01 (1.36, 2.98)	2.87 (1.79, 4.82)	2.98 (1.92, 4.79)

Above summary contains median and interquartile range of area under the curve values.

## Data Availability

Data from the HOME Study can be requested at https://homestudy.research.cchmc.org/contact.

## Appendix A. Supplementary data

Supplementary data to this article can be found online at https://doi.org/10.1016/j.envres.2026.124553.
