# Supplementary material for: Association between gestational and childhood exposures to organophosphate esters and social skill, problem behavior among adolescents
Source: Environ Res. Author manuscript; Available in PMC 2026 Jun 15. (PMC13267135; doi:10.1016/j.envres.2026.124553)
Supplement: 1 [file NIHMS2183704-supplement-1.docx]

Association between Gestational and Childhood Exposures to Organophosphate Esters with Social Skill, Problem Behavior among Adolescents

Jagadeesh Puvvula*, Joseph M. Braun, Kimberly Yolton, Kim M. Cecil, Antonia M. Calafat, Maria Ospina, Whitney Fitts, Kelli M. Williams, Weili Yang, Ann Vuong, Bruce Lanphear, Aimin Chen

*Corresponding author: Jagadeesh Puvvula - Department of Biostatistics, Epidemiology and Informatics, Perelman School of Medicine, University of Pennsylvania, Philadelphia, PA; email: [Jagadeesh.Puvvula@pennmedicine.upenn.edu](mailto:Jagadeesh.Puvvula@pennmedicine.upenn.edu)

Contents (11 pages): 8 tables and 4 figures

**Table of contents**

Table S1. Scales, Subscales, and Number of Survey Items for the SSiS……………….……..2

Table S2. Participants included for urinary OPE biomarker measurements..………………….2

Table S3. Frequency of OPE measures…………………………………………...……………….2

Table S4. Summary of urinary OPE biomarkers …………………………………...……….…….3

Table S5. Interclass correlation of OPE biomarkers…………………………..........…………….4

Table S6. Joint association between gestational, childhood, and lifetime measures of urinary OPE metabolites with social skill and problem behaviors among adolescents………………..5

Table S7. Interaction p-values by adolescent sex………………………………………………...6

Table S8. Associations between single OPE biomarkers and adolescent SSiS scores………7

Figure S1. Directed acyclic graph conceptualizing associations between OPE biomarkers and social skills improvement system…………………………………………………….………..8

Figure S2. Detection pattern of urinary OPE biomarkers measured during nine follow-up visits..…………………………………………………………………………………………………..9

Figure S3. Patterns of repeated OPE biomarker measures. ……………..……………………10

Figure S4. Comparison of self and caregiver-reported SSiS scores……………………..……11

| **Table S1. Scales, Subscales, and Number of Survey Items for the SSiS** | | |
| --- | --- | --- |
| **Scales and Subscales** | **Adolescent Items** | **Caregiver Items** |
| Social Skills Scale^a^ | 46 | 46 |
| Communication | 6 | 7 |
| Cooperation | 7 | 6 |
| Assertion | 7 | 7 |
| Responsibility | 7 | 6 |
| Empathy | 6 | 6 |
| Engagement | 7 | 7 |
| Self-Control | 6 | 7 |
| Problem Behaviors Scale^a^ | 29 | 33 |
| Externalizing | 12 | 12 |
| Bullying | 5 | 5 |
| Hyperactivity/Inattention | 7 | 7 |
| Internalizing | 10 | 10 |
| Autism Spectrum* | NA | 15 |
| ^a^-composite scores; *-recorded by caregivers only and includes questions from both social skills and problem behavior. | | |

| **Table S2. Participants included for urinary OPE biomarker measurements** | | | | |
| --- | --- | --- | --- | --- |
| Visit | # participants | AUC aggregation | | |
|  |  | Gestational | Childhood | Lifetime |
| 16 Weeks | 234 |  |  |  |
| 26 Weeks | 216 |  |  |  |
| Delivery | 209 |  |  |  |
| 1 Year | 165 |  |  |  |
| 2 Year | 150 |  |  |  |
| 3 Year | 161 |  |  |  |
| 5 Year | 160 |  |  |  |
| 8 Year | 191 |  |  |  |
| 12 Year | 225 |  |  |  |
| Aggregation of OPE biomarkers resulted in inclusion of 236 participants in gestational/lifetime exposure analysis and 232 participants in childhood analysis (Female=130 & Male=102). | | | | |

| **Table S3. Frequency of OPE measures** | | | |
| --- | --- | --- | --- |
| Gestation | Childhood | Lifetime | Count |
| <2 | <4 | <6 | 11 |
| <2 | ≥4 | ≥6 | 1 |
| ≥2 | <4 | <6 | 29 |
| ≥2 | <4 | ≥6 | 15 |
| ≥2 | ≥4 | ≥4 | 180 |
| Any measure | | | 236 |

| **Table S4. Summary of urinary OPE biomarkers** | | | | | |
| --- | --- | --- | --- | --- | --- |
| Biomarkers | Visit | N (%) | | | Median (IQR)^a^ |
|  |  | Not reported | Below LOD | Detected |  |
| **BCEP** | Gestational-16Weeks | 1 (0.43) | 27 (11.54) | 206 (88.03) | 0.59 (0.31, 1.07) |
|  | Gestational-26Weeks | 0 (0) | 35 (16.20) | 181 (83.80) | 0.52 (0.22, 1.21) |
|  | Delivery | 5 (2.39) | 22 (10.53) | 182 (89.72) | 0.67 (0.36, 1.26) |
|  | Child-1 Year | 2 (1.21) | 9 (5.45) | 154 (93.33) | 1.50 (0.71, 3.81) |
|  | Child-2 Year | 4 (2.67) | 14 (9.33) | 132 (88.00) | 1.18 (0.61, 3.25) |
|  | Child-3 Year | 0 (0) | 9 (5.59) | 152 (94.41) | 0.92 (0.38, 2.42) |
|  | Child-5 Year | 1 (0.62) | 9 (5.62) | 150 (93.75) | 0.64 (0.28, 1.46) |
|  | Child-8 Year | 3 (1.57) | 38 (19.90) | 150 (78.53) | 0.56 (0.26, 1.14) |
|  | Child-12 Year | 1 (0.44) | 65 (28.89) | 159 (79.63) | 0.47 (<LOD, 0.98) |
| **BDCIPP** | Gestational-16Weeks | 3 (1.28) | 7 (2.99) | 224 (95.73) | 0.70 (0.40, 1.40) |
|  | Gestational-26Weeks | 0 (0) | 22 (10.19) | 194 (89.81) | 0.70 (0.30, 1.10) |
|  | Delivery | 9 (4.31) | 15 (7.18) | 185 (88.52) | 0.80 (0.40, 1.60) |
|  | Child-1 Year | 0 (0) | 2 (1.21) | 163 (98.79) | 2.30 (1.20, 5.20) |
|  | Child-2 Year | 0 (0) | 1 (0.67) | 149 (99.33) | 2.50 (1.30, 5.50) |
|  | Child-3 Year | 0 (0) | 2 (1.24) | 159 (98.76) | 3.00 (1.30, 6.00) |
|  | Child-5 Year | 3 (1.88) | 1 (0.62) | 156 (97.50) | 2.50 (1.20, 5.70) |
|  | Child-8 Year | 0 (0) | 0 (0) | 191 (100) | 3.30 (1.60, 6.40) |
|  | Child-12 Year | 1 (0.44) | 1 (0.44) | 223 (99.11) | 2.50 (1.10, 4.60) |
| **DNBP** | Gestational-16Weeks | 2 (0.85) | 34 (14.53) | 198 (84.62) | 0.22 (0.15, 0.33) |
|  | Gestational-26Weeks | 4 (1.85) | 50 (23.15) | 162 (75.00) | 0.22 (0.13, 0.34) |
|  | Delivery | 15 (7.18) | 84 (40.19) | 110 (52.63) | 0.17 (<LOD, 0.26) |
|  | Child-1 Year* | 130 (78.79) | 0 (0) | 35 (21.21) | NA |
|  | Child-2 Year* | 147 (98.00) | 0 (0) | 3 (2.00) | NA |
|  | Child-3 Year | 35 (21.74) | 19 (11.80) | 107 (66.46) | 0.27 (0.15, 0.61) |
|  | Child-5 Year | 3 (1.88) | 25 (15.62) | 132 (82.50) | 0.22 (0.13, 0.35) |
|  | Child-8 Year | 0 (0) | 67 (35.08) | 124 (64.92) | 0.13 (<LOD, 0.21) |
|  | Child-12 Year | 1 (0.44) | 123 (54.67) | 101 (44.89) | <LOD (<LOD, 0.14) |
| **DPHP** | Gestational-16Weeks | 0 (0) | 2 (0.85) | 232 (99.15) | 1.50 (0.90, 2.70) |
|  | Gestational-26Weeks | 0 (0) | 4 (1.85) | 212 (98.15) | 1.50 (0.90, 2.50) |
|  | Delivery | 5 (2.39) | 1 (0.48) | 203 (97.13) | 2.10 (1.20, 4.10) |
|  | Child-1 Year | 1 (0.61) | 0 (0) | 164 (99.39) | 3.40 (2.00, 6.00) |
|  | Child-2 Year | 1 (0.67) | 0 (0) | 149 (99.33) | 3.20 (1.90, 5.10) |
|  | Child-3 Year | 0 (0) | 1 (0.62) | 160 (99.38) | 2.30 (1.50, 4.00) |
|  | Child-5 Year | 0 (0) | 0 (0) | 160 (100) | 2.10 (1.20, 5.10) |
|  | Child-8 Year | 0 (0) | 0 (0) | 191 (100) | 1.90 (1.10, 3.30) |
|  | Child-12 Year | 1 (0.44) | 1 (0.44) | 223 (97.13) | 1.80 (1.10, 3.40) |
| *-Excluded for analysis in this study. a-specific gravity standardized OPE biomarker concentrations measured as μg/L. Median and IQR were calculated up on left-truncated imputation of observations <LOD. Summary at childhood visits at age 1 & 2 years were not reported due to relatively lower number of study participants with DNBP biomarker measurements. | | | | | |

| **Table S5. Interclass correlation of OPE biomarkers** | | | |
| --- | --- | --- | --- |
| OPE biomarker | Gestation | Childhood | Lifetime |
| BCEP | 0.28 | 0.11 | 0.10 |
| BDCIPP | 0.35 | 0.19 | 0.11 |
| DNBP | 0.21 | 0.09 | 0.04 |
| DPHP | 0.07 | 0.02 | 0.07 |
| Intraclass correlation coefficients (ICCs) were calculated using linear mixed-effects models. For each analyte, a model with a random intercept for participant was fit to the log2-transformed specific gravity standardized OPE biomarker concentrations using restricted maximum likelihood (REML) estimation. ICC = σ²between / (σ²between + σ²within). | | | |

| **Table S6. Joint association between gestational, childhood, and lifetime measures of urinary OPE metabolites with social skill and problem behaviors among adolescents** | | | | | | | | |
| --- | --- | --- | --- | --- | --- | --- | --- | --- |
|  | *Ψ_bootstrap_* (95%CI) | *Ψ* (95%CI) | +*Ψ* | -*Ψ* | OPE biomarker weight | | | |
|  |  |  |  |  | BCEP | BDCIPP | DNBP | DPHP |
| Adolescent reported – Problem Behavior – Overall | | | | | | | | |
| Gestational | 1.06 (-0.88, 2.99) | 1.06 (-1.05, 3.16) | 2.51 | -1.46 | 0.38 | -1 | 0.38 | 0.24 |
| Childhood* | -0.37 (-2.78, 2.05) | -0.37 (-2.45, 1.72) | 0.54 | -0.91 | -0.44 | -0.08 | 1 | -0.48 |
| Lifetime* | -0.63 (-2.86, 1.60) | -0.63 (-2.71, 1.45) | 0.3 | -0.93 | -0.05 | -0.27 | 1 | -0.68 |
| Adolescent reported – Problem Behavior - Female | | | | | | | | |
| Gestational | 1.49 (-1.11, 4.09) | 1.49 (-1.36, 4.34) | 2.21 | -0.72 | 0.58 | -1 | 0.41 | 0.02 |
| Childhood | -3.40 (-6.89, 0.10) | -3.40 (-6.37, -0.42) | 0.14 | -3.53 | -0.4 | 1 | -0.3 | -0.29 |
| Lifetime | -3.45 (-6.95, 0.05) | -3.45 (-6.25, -0.64) | 0 | -3.45 | -0.2 | -0.12 | -0.3 | -0.38 |
| Adolescent reported – Problem Behavior - Male | | | | | | | | |
| Gestational | 0.87 (-2.67, 4.41) | 0.87 (-2.39, 4.12) | 3.39 | -2.53 | 0.35 | -1 | 0.38 | 0.27 |
| Childhood | 2.14 (-1.32, 5.60) | 2.14 (-0.96, 5.23) | 2.56 | -0.42 | 0.15 | -1 | 0.66 | 0.18 |
| Lifetime | 2.21 (-0.88, 5.31) | 2.21 (-0.81, 5.24) | 2.68 | -0.46 | -0.19 | -0.81 | 0.82 | 0.18 |
| Caregiver reported – Problem Behavior – Overall | | | | | | | | |
| Gestational | -1.19 (-2.69, 0.31) | -1.19 (-2.91, 0.52) | 0.31 | -1.5 | 1 | -0.03 | -0.67 | -0.3 |
| Childhood* | -1.51 (-3.17, 0.16) | -1.51 (-3.13, 0.11) | 0.84 | -2.34 | -0.45 | -0.54 | -0.01 | 1 |
| Lifetime* | -1.47 (-3.20, 0.26) | -1.47 (-3.12, 0.18) | 0.88 | -2.35 | -0.25 | -0.75 | 0.25 | 0.75 |
| Caregiver reported – Problem Behavior - Female | | | | | | | | |
| Gestational | -0.82 (-3.18, 1.53) | -0.82 (-3.22, 1.58) | 0.51 | -1.33 | -0.46 | 0.74 | -0.54 | 0.26 |
| Childhood | -3.44 (-6.05, -0.84) | -3.44 (-5.86, -1.03) | 0.57 | -4.01 | -0.61 | -0.22 | -0.17 | 1 |
| Lifetime | -3.82 (-6.43, -1.22) | -3.82 (-6.06, -1.59) | 0.72 | -4.54 | -0.55 | -0.27 | -0.18 | 1 |
| Caregiver reported – Problem Behavior - Male | | | | | | | | |
| Gestational | -1.79 (-4.23, 0.66) | -1.79 (-4.25, 0.68) | 1.51 | -3.3 | 1 | -0.52 | -0.29 | -0.19 |
| Childhood | 1.22 (-1.01, 3.45) | 1.22 (-0.98, 3.43) | 2.86 | -1.63 | 0.33 | -1 | 0.41 | 0.25 |
| Lifetime | 0.62 (-1.56, 2.81) | 0.62 (-1.62, 2.86) | 3.52 | -2.9 | 0.5 | -1 | 0.33 | 0.17 |
| Adolescent reported – Social skill – Overall | | | | | | | | |
| Gestational | -0.92 (-3.65, 1.82) | -0.92 (-3.59, 1.76) | 0.9 | -1.82 | -0.34 | 0.78 | -0.66 | 0.22 |
| Childhood* | -1.18 (-3.90, 1.55) | -1.18 (-3.79, 1.43) | 0.18 | -1.36 | -0.48 | 0.93 | -0.52 | 0.07 |
| Lifetime* | -1.53 (-4.20, 1.13) | -1.53 (-4.15, 1.09) | 0.02 | -1.55 | -0.54 | -0.14 | -0.32 | 1 |
| Adolescent reported – Social skill - Female | | | | | | | | |
| Gestational | -0.95 (-5.14, 3.25) | -0.95 (-4.79, 2.89) | 0.64 | -1.59 | -0.96 | 0.44 | -0.04 | 0.56 |
| Childhood | 1.38 (-2.94, 5.71) | 1.38 (-2.66, 5.43) | 2.47 | -1.08 | 0.34 | 0.48 | 0.18 | -1 |
| Lifetime | 1.00 (-3.09, 5.09) | 1.00 (-2.83, 4.84) | 1.65 | -0.64 | -0.76 | 0.84 | -0.24 | 0.16 |
| Adolescent reported – Social skill - Male | | | | | | | | |
| Gestational | -1.17 (-4.64, 2.30) | -1.17 (-4.95, 2.61) | 1.19 | -2.36 | 0.36 | 0.64 | -0.82 | -0.18 |
| Childhood | -2.88 (-6.39, 0.63) | -2.88 (-6.36, 0.60) | 0 | -2.88 | -0.54 | -0.23 | -0.21 | -0.03 |
| Lifetime | -3.59 (-6.79, -0.40) | -3.59 (-7.02, -0.17) | 0 | -3.59 | -0.31 | -0.42 | -0.17 | -0.1 |
| Caregiver reported – Social skill – Overall | | | | | | | | |
| Gestational | 1.79 (-0.85, 4.43) | 1.79 (-0.72, 4.30) | 2.2 | -0.41 | 0.33 | 0.5 | 0.18 | -1 |
| Childhood | -0.47 (-3.11, 2.16) | -0.47 (-2.94, 1.99) | 1.45 | -1.92 | 0.31 | 0.69 | -0.08 | -0.92 |
| Lifetime | 0.01 (-2.73, 2.75) | 0.01 (-2.45, 2.47) | 1.41 | -1.4 | 0.44 | 0.35 | 0.22 | -1 |
| Caregiver reported – Social skill - Female | | | | | | | | |
| Gestational | 2.40 (-1.23, 6.04) | 2.40 (-0.90, 5.71) | 2.94 | -0.54 | 0.73 | 0.1 | -1 | 0.18 |
| Childhood | -0.65 (-4.53, 3.23) | -0.65 (-4.19, 2.89) | 1.41 | -2.06 | 0.95 | 0.05 | -0.18 | -0.82 |
| Lifetime | -0.38 (-4.44, 3.69) | -0.38 (-3.70, 2.95) | 1.62 | -1.99 | 1 | -0.28 | -0.21 | -0.52 |
| Caregiver reported – Social skill - Male | | | | | | | | |
| Gestational | 1.95 (-2.06, 5.95) | 1.95 (-1.85, 5.74) | 4.42 | -2.47 | -0.48 | 0.65 | 0.35 | -0.52 |
| Childhood | -1.42 (-5.67, 2.83) | -1.42 (-5.09, 2.25) | 1.44 | -2.86 | -0.48 | 1 | -0.03 | -0.49 |
| Lifetime | -0.38 (-4.17, 3.42) | -0.38 (-3.94, 3.18) | 2.77 | -3.15 | -0.32 | 0.74 | 0.26 | -0.68 |
| *Ψ* – interpreted as every quartile increase in OPE biomarker mixture associated with SSiS scores. Associations adjusted for adolescent sex (for overall analysis), race, caregiver marital status, maternal education, maternal age at delivery, maternal depression, household income, blood lead, serum cotinine, and Relational Frustration scores. *Statistically significant interaction (p<0.1) between OPE biomarker mixture and adolescent sex, refer table S4 for p-values. OPE biomarker weights contributing to -*Ψ* were multiplied by -1 for presentation purposes. | | | | | | | | |

| **Table S7. Interaction p-values by adolescent sex** | | |
| --- | --- | --- |
|  | *p-value bootstrap* | *p-value non-bootstrap* |
| **Adolescent reported – Problem Behavior** | | |
| Gestational | 0.55 | 0.54 |
| Childhood | 0.02* | 0.01* |
| Lifetime | 0.03* | 0.02* |
| **Caregiver reported – Problem Behavior** | | |
| Gestational | 0.18 | 0.22 |
| Childhood | 0.01* | 0.01* |
| Lifetime | 0.01* | 0.01* |
| **Adolescent reported – Social skill** | | |
| Gestational | 0.77 | 0.75 |
| Childhood | 0.06* | 0.07* |
| Lifetime | 0.06* | 0.06* |
| **Caregiver reported – Social skill** | | |
| Gestational | 0.98 | 0.97 |
| Childhood | 0.80 | 0.78 |
| Lifetime | 0.74 | 0.71 |
| Interaction p-values (biomarker mixture and adolescent sex) obtained using the quantile g-computation approach. *Indicates statistical significance | | |

| **Table S8. Associations between single OPE biomarkers and adolescent SSiS scores** | | | | |
| --- | --- | --- | --- | --- |
|  | BCEP | BDCIPP | DNBP | DPHP |
| **Adolescent reported – Problem Behavior – Overall** | | | | |
| Gestational | 0.61 (-0.30, 1.53) | -0.77 (-1.88, 0.33) | 0.70 (-0.70, 2.09) | 0.26 (-1.02, 1.55) |
| Childhood | -0.24 (-1.06, 0.58) | -0.52 (-1.74, 0.70) | 0.11 (-1.30, 1.51) | -0.55 (-1.99, 0.89) |
| Lifetime | -0.10 (-1.08, 0.88) | -0.59 (-1.77, 0.59) | 0.26 (-1.16, 1.69) | -0.73 (-2.25, 0.79) |
| **Adolescent reported – Problem Behavior - Female** | | | | |
| Gestational | 0.68 (-0.47, 1.83) | -0.13 (-1.58, 1.31) | 0.52 (-1.38, 2.43) | -0.10 (-1.64, 1.43) |
| Childhood | -1.01 (-2.14, 0.13) | -1.05 (-2.66, 0.56) | -1.32 (-3.21, 0.56) | -1.19 (-3.02, 0.64) |
| Lifetime | -0.86 (-2.24, 0.51) | -1.22 (-2.79, 0.36) | -1.29 (-3.20, 0.63) | -1.20 (-3.09, 0.68) |
| **Adolescent reported – Problem Behavior - Male** | | | | |
| Gestational | 0.53 (-1.08, 2.14) | -2.03 (-3.86, -0.20) | 0.68 (-1.48, 2.85) | 0.81 (-1.70, 3.33) |
| Childhood | 0.35 (-0.90, 1.61) | -0.07 (-2.06, 1.92) | 2.05 (-0.21, 4.31) | 0.24 (-2.28, 2.75) |
| Lifetime | 0.55 (-0.91, 2.02) | -0.07 (-1.95, 1.82) | 2.32 (0.06, 4.58) | -0.24 (-2.96, 2.49) |
| **Caregiver reported – Problem Behavior – Overall** | | | | |
| Gestational | -0.11 (-0.86, 0.63) | -0.70 (-1.60, 0.20) | -1.05 (-2.17, 0.07) | -0.70 (-1.74, 0.34) |
| Childhood | -0.55 (-1.20, 0.09) | -1.03 (-2.00, -0.07) | 0.04 (-1.07, 1.16) | 0.36 (-0.78, 1.51) |
| Lifetime | -0.65 (-1.44, 0.14) | -1.51 (-2.45, -0.57) | -0.21 (-1.36, 0.94) | -0.10 (-1.34, 1.13) |
| **Caregiver reported – Problem Behavior - Female** | | | | |
| Gestational | -0.39 (-1.36, 0.57) | -0.48 (-1.68, 0.73) | -0.95 (-2.53, 0.64) | -0.64 (-1.92, 0.64) |
| Childhood | -1.20 (-2.13, -0.26) | -1.36 (-2.69, -0.03) | -0.53 (-2.12, 1.05) | -0.58 (-2.12, 0.96) |
| Lifetime | -1.83 (-2.94, -0.72) | -1.77 (-3.06, -0.48) | -0.93 (-2.54, 0.67) | -1.08 (-2.66, 0.49) |
| **Caregiver reported – Problem Behavior - Male** | | | | |
| Gestational | 0.50 (-0.74, 1.73) | -1.55 (-2.96, -0.15) | -0.99 (-2.64, 0.66) | -0.97 (-2.89, 0.95) |
| Childhood | 0.30 (-0.60, 1.20) | -0.43 (-1.86, 1.00) | 1.35 (-0.28, 2.99) | 2.01 (0.25, 3.77) |
| Lifetime | 0.70 (-0.42, 1.81) | -1.05 (-2.48, 0.38) | 1.39 (-0.36, 3.14) | 1.54 (-0.53, 3.61) |
| **Adolescent reported – Social skill – Overall** | | | | |
| Gestational | -0.73 (-1.89, 0.42) | 0.64 (-0.76, 2.03) | -0.51 (-2.27, 1.25) | 0.51 (-1.11, 2.13) |
| Childhood | -0.76 (-1.78, 0.26) | -0.10 (-1.63, 1.44) | -0.93 (-2.68, 0.83) | -1.00 (-2.80, 0.80) |
| Lifetime | -0.97 (-2.20, 0.27) | -0.18 (-1.68, 1.31) | -0.90 (-2.69, 0.89) | -0.76 (-2.68, 1.17) |
| **Adolescent reported – Social skill - Female** | | | | |
| Gestational | -1.14 (-2.67, 0.39) | 0.37 (-1.57, 2.30) | 0.58 (-1.97, 3.13) | 0.95 (-1.11, 3.00) |
| Childhood | -0.22 (-1.75, 1.31) | 1.22 (-0.93, 3.37) | -0.52 (-3.05, 2.02) | -1.01 (-3.46, 1.44) |
| Lifetime | -0.47 (-2.32, 1.39) | 1.36 (-0.75, 3.47) | -0.10 (-2.68, 2.49) | -0.22 (-2.77, 2.32) |
| **Adolescent reported – Social skill - Male** | | | | |
| Gestational | 0.29 (-1.55, 2.13) | 1.04 (-1.08, 3.17) | -1.58 (-4.03, 0.87) | -0.84 (-3.70, 2.02) |
| Childhood | -1.41 (-2.79, -0.03) | -1.73 (-3.95, 0.48) | -1.16 (-3.74, 1.43) | -1.00 (-3.83, 1.83) |
| Lifetime | -1.49 (-3.14, 0.15) | -1.96 (-4.07, 0.15) | -1.62 (-4.23, 0.99) | -1.55 (-4.64, 1.54) |
| **Caregiver reported – Social skill – Overall** | | | | |
| Gestational | 0.57 (-0.51, 1.66) | 1.23 (-0.08, 2.54) | 1.26 (-0.39, 2.91) | -0.08 (-1.60, 1.45) |
| Childhood | 0.01 (-0.96, 0.98) | 0.65 (-0.80, 2.11) | -1.02 (-2.69, 0.64) | -1.34 (-3.04, 0.37) |
| Lifetime | 0.08 (-1.08, 1.25) | 0.83 (-0.57, 2.24) | -0.14 (-1.83, 1.54) | -1.66 (-3.46, 0.14) |
| **Caregiver reported – Social skill - Female** | | | | |
| Gestational | 0.84 (-0.50, 2.19) | 0.80 (-0.88, 2.48) | -0.40 (-2.63, 1.83) | 0.10 (-1.69, 1.90) |
| Childhood | -0.15 (-1.49, 1.19) | 0.05 (-1.85, 1.95) | -1.48 (-3.70, 0.73) | -1.44 (-3.59, 0.70) |
| Lifetime | 0.53 (-1.09, 2.14) | 0.04 (-1.82, 1.90) | -0.79 (-3.04, 1.46) | -1.17 (-3.38, 1.04) |
| **Caregiver reported – Social skill - Male** | | | | |
| Gestational | -0.10 (-2.01, 1.80) | 2.53 (0.38, 4.68) | 2.98 (0.50, 5.46) | -0.37 (-3.33, 2.60) |
| Childhood | -0.17 (-1.65, 1.31) | 0.92 (-1.42, 3.27) | -1.32 (-4.02, 1.39) | -2.29 (-5.22, 0.64) |
| Lifetime | -0.59 (-2.32, 1.13) | 1.35 (-0.86, 3.55) | 0.08 (-2.64, 2.81) | -3.13 (-6.27, 0.02) |
| Effect estimates and 95% confidence intervals in this table generated using multiple linear regression. Effect estimates interpreted as every 2-fold increase in OPE biomarker is associated with SSiS scores, adjusted for maternal education, race, marital status, age at delivery, depression, relationship frustration score, household income, blood lead, and serum cotinine. | | | | |


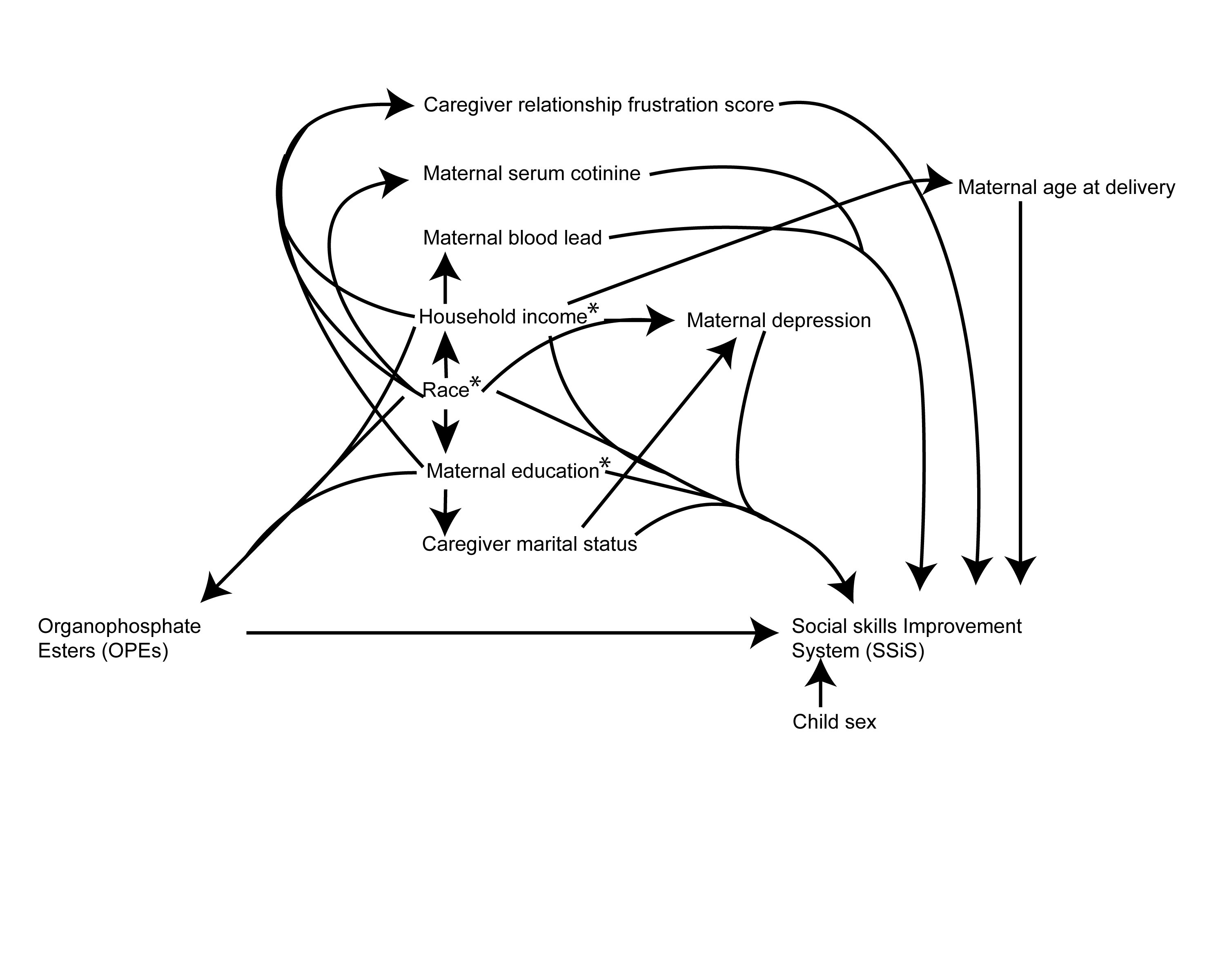


Figure S1. Directed acyclic graph conceptualizing associations between organophosphate ester biomarkers and the social skills improvement system. The covariates listed here were selected based on the literature. *Covariates that are within the causal pathway.


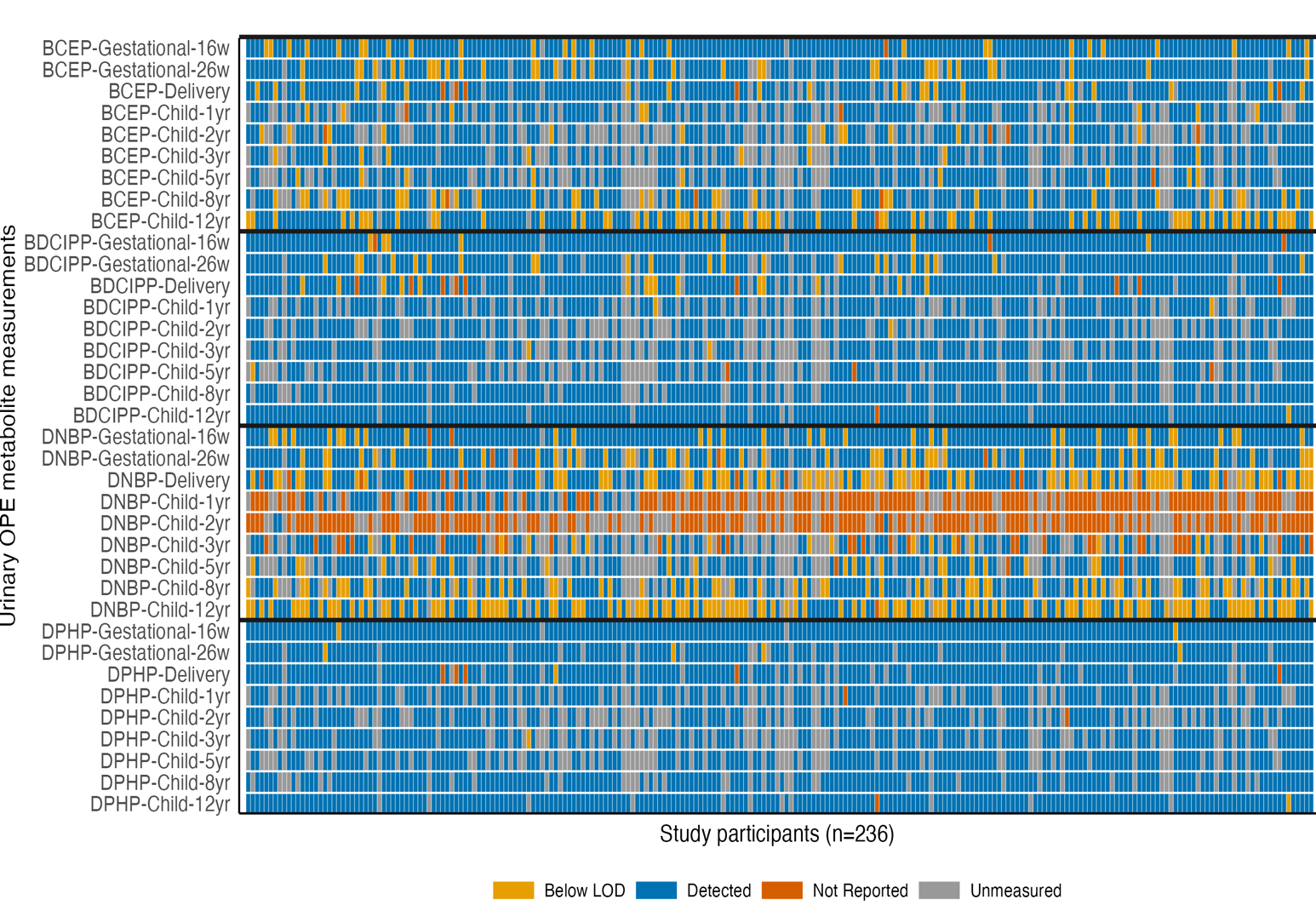


Figure S2. Urinary OPE biomarker patterns measured during nine follow-up visits. The X-axis represents the study participants. The Y-axis includes OPE biomarkers by visit. Unmeasured refers to study participants who did not provide urine samples during a follow-up visit.

| 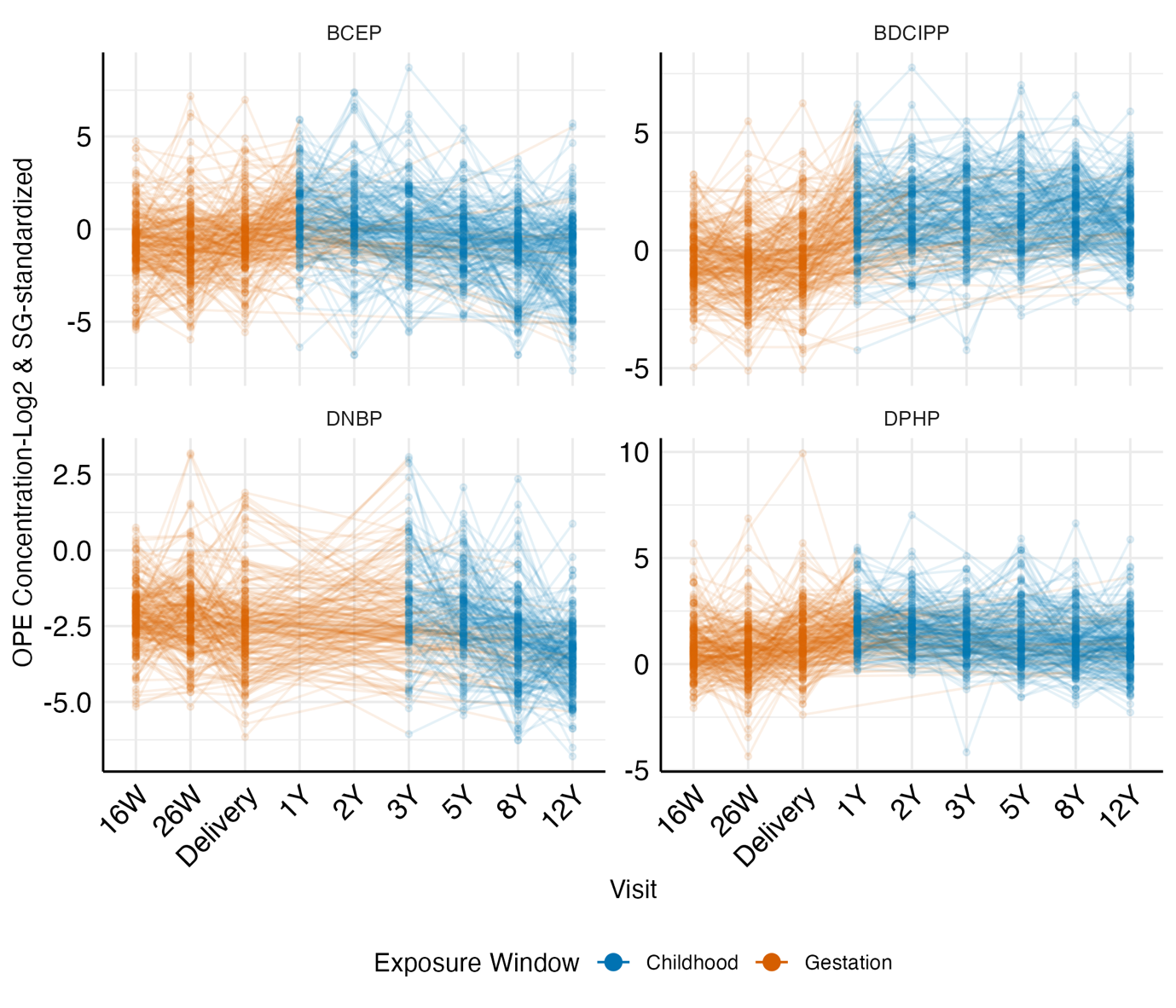 |
| --- |
| Figure S3. Patterns of repeated OPE biomarker measures. |

| 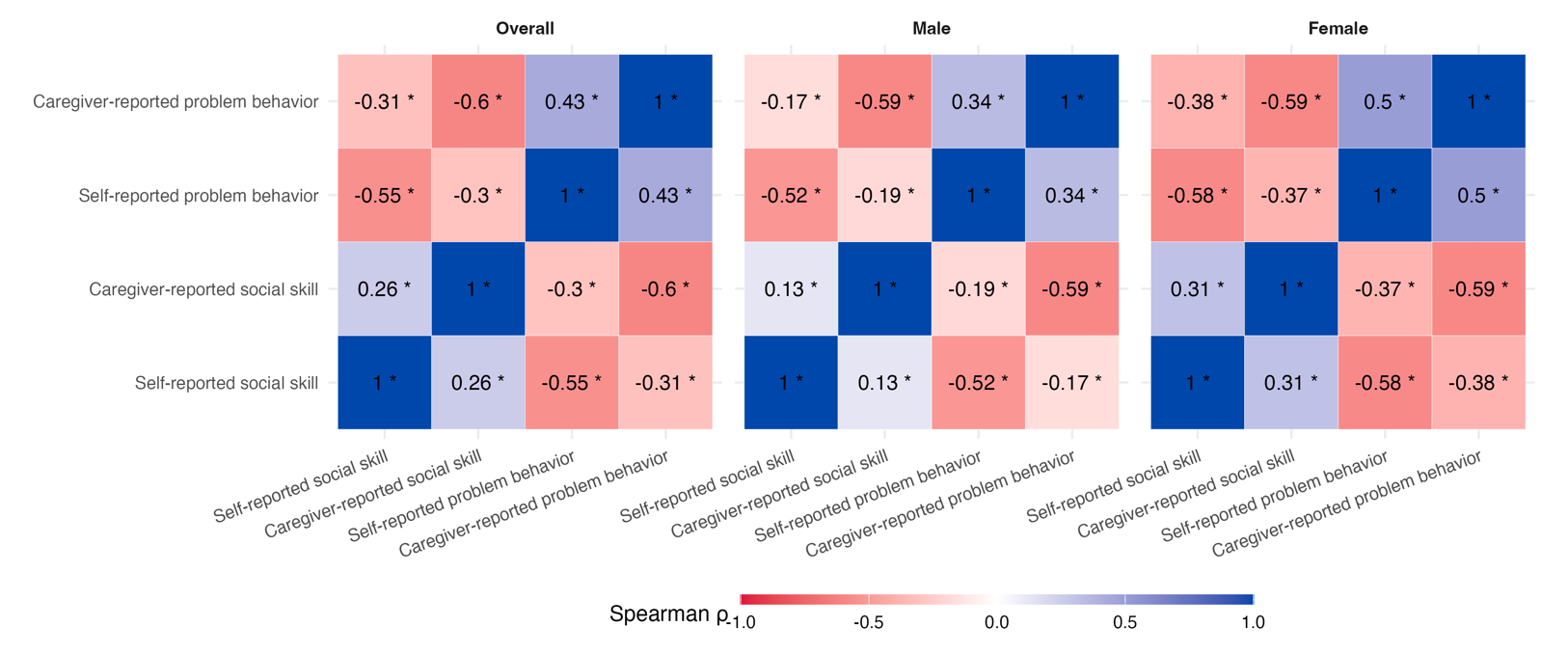  **A** |
| --- |
| 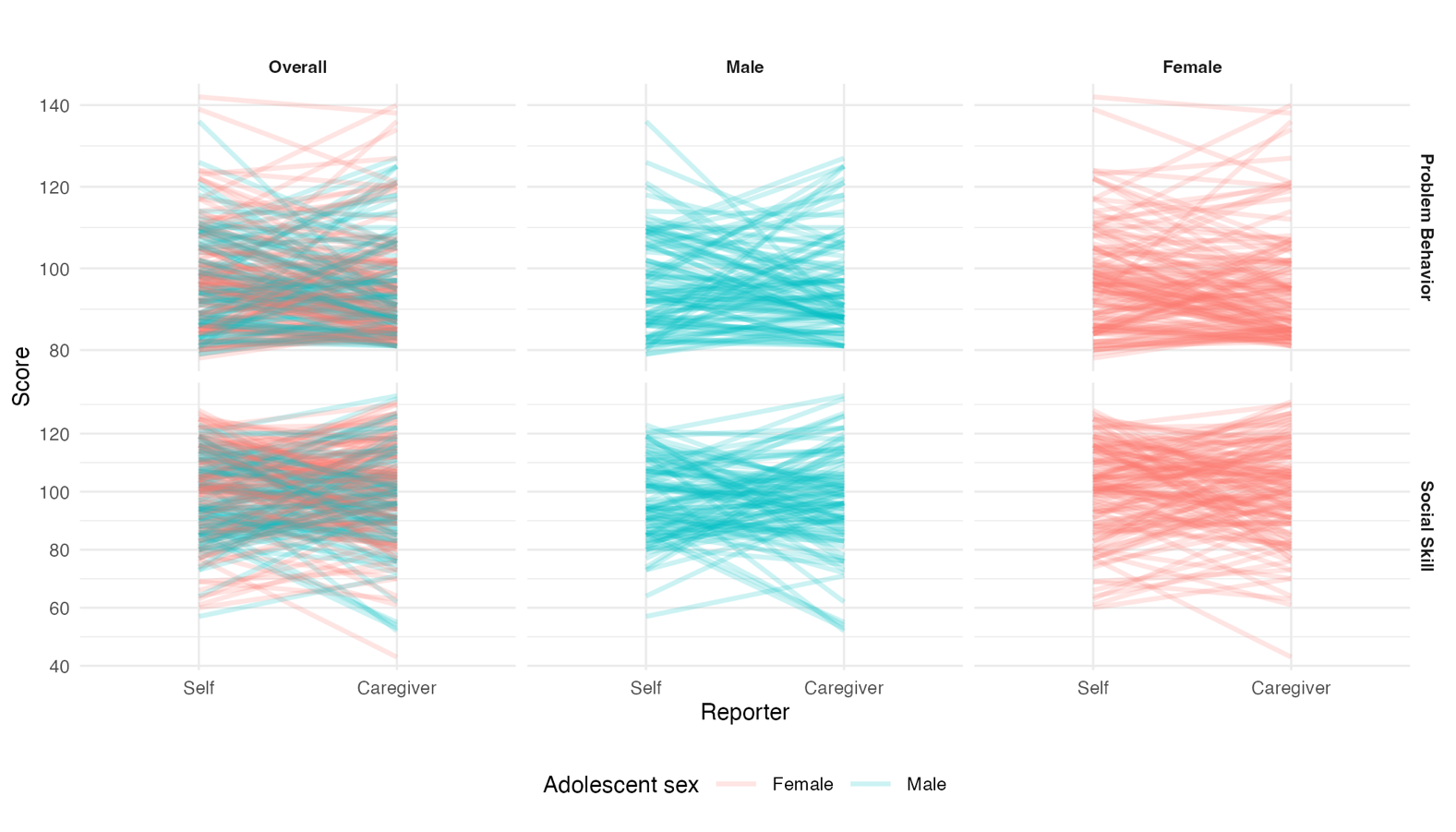  B |
| Figure S4. Comparison of self and caregiver reported SSiS scores. A-Spearman correlation coefficients between self-reported and caregiver reported SSiS scores. Blue color palate indicates positive correlation and red indicates negative correlation. *Statistical significance using p-value 0.05 as threshold. B-Potential differences in SSiS scores between self and caregiver reported. Solid lines represent median score trends by gender. |
